# Facing the pandemic aftermath: exploring depression and mental health service usage among first-year university students in southern Thailand

**DOI:** 10.3389/fpsyt.2025.1555361

**Published:** 2025-08-20

**Authors:** Waluwan Pratummanee, Tharntip Sangsuwan, Chonnakarn Jatchavala, Thanitha Sirirak

**Affiliations:** ^1^ Department of Family Medicine and Preventive Medicine, Faculty of Medicine, Prince of Songkla University, Hatyai, Songkhla, Thailand; ^2^ Department of Psychiatry, Faculty of Medicine, Prince of Songkla University, Hatyai, Songkhla, Thailand

**Keywords:** depression, students, mental health service, cross-sectional studies, COVID-19

## Abstract

**Introduction:**

Depression is a serious mental health concern in Thailand and a leading cause of psychological and physical impairment. Untreated depression can progress to more severe disorders. University students, in particular, must navigate developmental stages and pedagogical approaches to higher education curricula. Without appropriate intervention, depression in this population may progress to more severe depressive disorders. This study examined the prevalence, associated factors, and health service utilization patterns among first-year university students experiencing depression.

**Methods:**

This cross-sectional study was conducted using an online survey among first-year students in southern Thailand three months into the semester, following the government’s approval to transition COVID-19 to an endemic disease in 2022. The survey collected data on demographic information, factors associated with depression, and mental health services access behavior. Descriptive statistics and logistic regression analysis were performed for data analysis.

**Results:**

A total of 1,611 students participated in the study, 80.3% of whom were female, with an age range of 18–20 years (mean: 19.4, SD: 1.12). The prevalence of depression was 37.5%, with severity levels distributed as follows: moderate (21.4%), moderate to severe (11.4%), and severe (4.7%). Protective factors against depression included a grade point average (GPA) of 2.00–2.49 or 4.00, attending private schools, being single, having close relationships with friends and partners, and following a healthy diet, and moderate to high self-confidence was associated with an increased likelihood of depression. Most participants did not consider seeing a psychiatrist troublesome or embarrassing, either for themselves or others. Moreover, they did not perceive the process of accessing mental health services in Thailand as complicated. However, they did not tend to seek mental health information online or from friends, teachers, parents or guardians.

**Discussion:**

The prevalence of depression among participants from southern universities in Thailand that are members of the ASEAN University Network–Health Promotion Network during the first three months of the academic year was 37.5%. Protective and risk factors included GPA, high school type, presence of a partner, relationships with friends and partners, eating habits, and self-confidence. Although students generally perceived mental health services as accessible, many were unaware of university-provided services and did not seek mental health information from close contacts such as parents, guardians, teachers, or friends.

## 1 Introduction

Depression is a mental disorder often triggered by a stressful life event or bereavement. In some instances, it may arise from perceived events, anticipations, or personal cognitions. When real events precede expected thoughts, individuals may experience excessive or prolonged grief (1). Untreated prolonged grief can result in major depressive disorders. Depression is a prevalent concern in Thailand and can significantly impact physical health. According to the World Health Organization (2), approximately 322 million individuals globally, or 4.4% of the global population, suffered from depression in 2017.

In Thailand, a significant proportion of individuals with depression remain undiagnosed or untreated (3). A 2019 report on depression care services revealed that only 69.1% of diagnosed patients received treatment. Epidemiological surveys have indicated that 11.5% of Thai individuals aged 15–24 years are diagnosed with depression, with university students identified as a high-risk group owing to substantial developmental and academic adjustments (4). This transitional age involves changes such as shifting from online to onsite learning post-COVID. Before the COVID-19 pandemic, higher education in Thailand was primarily conducted onsite (5). The shift to online learning introduced many students to flexible home-based routines. Returning to onsite classes required stricter schedules and renewed social interactions, causing many students to struggle with readjustment and feel isolated. Estimated prevalence rates of depression among students range from 16.7% to 52.5% internationally (6, 7) and from 20% to 38.81% within Thailand (8, 9) during the COVID-19 pandemic (10). These findings suggest that the COVID-19 pandemic exacerbated the number of students experiencing depression. Furthermore, students experienced challenges related to peer and romantic relationships, career choices, and adult responsibilities (11), further amplifying their vulnerability to depression in the absence of appropriate treatment or interventions.

According to 2022 data on suicide issues in Thailand from the Suicide Surveillance Center at Khon Kaen Rajanagarindra Psychiatric Hospital, adolescents and students aged 10–19 years had the highest rate of suicide attempts among all age groups, at 224.34 per 100,000 population. The majority of individuals in this group were female (73.2%), and 44.6% were students. The primary reported cause of suicide attempts was mental health problems (12). In 2018, a study conducted by the Child and Adolescent Mental Health Rajanagarindra Institute on the prevalence of depression and suicide risk among adolescents aged 10–19 years (n = 5,345) found that 22.5% were at risk of suicide, and those at risk were 9.8 times more likely to experience depression compared to adolescents without suicide risk (13). Early detection of depression allows for prompt intervention, which can reduce the severity of symptoms and prevent the condition from progressing into a chronic or more severe state (14). Timely treatment also reduces the risk of complications such as substance abuse, suicidal ideation, and related physical health issues. Moreover, it facilitates faster recovery, enabling individuals to more quickly return to their academic, occupational, and social roles.

The ASEAN University Network–Health Promotion Network (AUN-HPN) brings together expertise from regional educational institutions to establish “Healthy Universities” by encouraging positive lifestyles and creating supportive environments. This initiative strengthens the role of universities in fostering well-being and developing conditions that support holistic development. These institutions are defined as those that actively advance overall quality of life across physical, mental, social, and environmental dimensions. This collaboration involves 30 higher education institutions across 10 ASEAN countries and aims to improve the quality of life for individuals in the Southeast Asian region through cooperative mechanisms in health promotion activities. In southern Thailand, where ongoing unrest in the border provinces has contributed to mental health challenges, studies have shown that 30.8% of students experience post-traumatic stress disorder (PTSD) (15). Research also indicates that PTSD often co-occurs with depression (16), suggesting the presence of underlying psychological issues in the region—likely intensified by the COVID-19 pandemic. In response, Prince of Songkla University, as the main driver of the AUN-HPN network in the South, is actively working to strengthen the university network in the region, with the long-term goal of establishing Healthy Universities.

This study examined the prevalence of depression over a three-month period during students’ first academic year at southern AUN-HPN network universities in Thailand. Additionally, factors associated with depression and students’ mental health services utilization behavior were examined. Conducted after the declaration of the end of the COVID-19 pandemic, the identification of depression prevalence and its associated risk factors provides essential insights into the mental health status of the target population. It contributes to a better understanding of the severity of the problem among a specific age group, and serves as a foundation for effective planning and resource allocation. Moreover, relevant agencies and organizations can implement targeted and appropriate preventive measures to reduce risks more precisely. This approach may also help to mitigate the long-term impact on students’ mental health and promote their overall well-being throughout their academic journey and into their future lives.

## 2 Materials and methods

### 2.1 Study design and participants

A cross-sectional study design was employed, and 1,900 first-year students from southern AUN-HPN network universities in Thailand were recruited as participants. The sample size was calculated using the finite population proportion formula with the following parameters: Proportion (p) = 0.685 (17), Alpha (α) = 0.05, Error (d) = 0.05, and an estimated non-response rate of 10%. Data were collected during the first academic semester, three months after the semester commencement (September 2022–December 2022), following the government’s declaration of the end of the COVID-19 pandemic (18).

### 2.2 Measures

This study used a self-administered questionnaire, comprising three sections. Part 1 covered the baseline characteristics and factors associated with depression, including age, sex, religion, chronic illness, residential area, family history of mental illness, alcohol consumption, smoking, substance use, sleep adequacy, physical activity, dietary habits, presence of a stable partner, monthly income, time management, faculty, grade point average (GPA), high school type, faculty relevance to future career, self-esteem, daily internet usage, close relationships, and life satisfaction. Part 2 comprised the Patient Health Questionnaire (PHQ-9) for assessing depression, which was translated into a Thai version (19). The Thai version demonstrated a reliability coefficient of 0.79 with a sensitivity of 85.3%, and specificity of 77.3% as a continuous measure. The questionnaire comprises nine items, with scores ranging between 0 and 27, and scores are interpreted as follows: 0–4 (none to minimal), 5–9 (mild), 10–14 (moderate), 15–19 (moderate to severe), and 20–27 (severe) (20). For the purposes of data analysis, scores between 0 and 9 indicated no depression, while scores of ≥10 indicated depression. Part 3 consisted of a questionnaire on access to mental health services, adapted from a study by Jampaklay et al. (21) on the migration of Thai Muslims to Malaysia and social integration with problem-solving in the three southern border provinces. This questionnaire addresses access to health services, well-being, satisfaction, and mental health. It contains nine items with responses categorized as “never” or “always,” reflecting participants’ perceptions of mental health services accessibility in Thailand. The instrument had a validity of 0.78 and a Cronbach’s alpha of 0.70.

### 2.3 Data collection

Data was collected using a Google Forms survey, accessible via a QR code provided to the participants. The survey targeted first-year students at universities in the southern AUN-HPN. All the universities are located in the southern region and are government-regulated institutions, sharing a common goal of developing into universities that promote health and well-being.

### 2.4 Data analysis

Descriptive statistics, including frequencies, percentages, means, and standard deviations, were used to summarize general characteristics such as age, sex, religion, chronic illness, residential area, family history of mental illness, alcohol consumption, smoking status, substance use, sleep adequacy, physical activity, dietary habits, presence of a stable partner, monthly income, time management, faculty affiliation, GPA, high school type, faculty relevance to future careers, self-esteem, daily internet usage, close relationships, and life satisfaction. The comparison of means or medians of continuous variables between groups with or without depression was performed using t-tests or the Wilcoxon rank sum test. Comparisons of categorical performed using the chi-squared test or Fisher’s exact test. A significance level of 0.05 was applied. Factors influencing depression (multivariable analysis) were analyzed using multiple logistic regression analysis.

## 3 Results

### 3.1 Sociodemographic characteristics

A total of 1,611 individuals participated in the study, with 80.3% being female. Participants’ ages ranged between 18 and 20 years (mean: 19.4, SD: 1.12). Most participants were students from lower-tier universities (77.2%) enrolled in the faculties of humanities and social sciences (68%). Most participants had an average GPA between 3.00 and 3.49 (38.1%). Regarding religious affiliation, 57.1% were Buddhists and 42.2% were Muslims. Monthly incomes were predominantly <5,000 baht (69.4%) The majority of participants (91.2%) reported no chronic illness history and 94.6% indicated no direct family history of psychiatric disorders. Most participants had completed their secondary education at public schools (71.6%) and predominantly resided in rural areas (65.4%). Regarding relationships, 47.4% had partners while 52.6% did not. Participants reported close relationships with friends (46.9%) and parents (32.2%). Most participants’ fields of study aligned with their intended future careers (93.2%). Moreover, 77% exhibited a medium level of self-confidence, and 40.5% and 45.3% reported moderate and high life satisfaction, respectively, (**Table 1**).

**Table 1:** Participants’ sociodemographic characteristics stratified by depression (n =1611).

Variable	Depression		Total	*P*-value
	No	Yes		
Sex, n (%)				0.862^a^
Female	881 (80.5)	483 (80.1)	1294 (80.3)	
Male	197 (19.5)	120 (19.9)	317 (19.7)	
Age (years), n (%)				0.008^a^
18–20	848 (84.1)	536 (88.9)	1384 (85.9)	
>20	160 (15.9)	63 (11.1)	227 (14.1)	
Religion, n (%)				0.283^a^
Buddhist	586 (58.1)	334 (55.4)	920 (57.1)	
Islam	414 (11.1)	266 (44.1)	680 (42.2)	
Christianity	4 (0.4)	3 (0.5)	7 (0.4)	
Did not specify	4 (0.4)	0 (0)	4 (0.2)	
University, n (%)				<0.001^a^
Upper southern region	368 (36.5)	0 (0)	368 (22.8)	
Lower southern region	640 (63.5)	603 (100)	1243 (77.2)	
Faculty, n (%)				<0.001^a^
Health Sciences	184 (18.3)	0 (0)	184 (11.4)	
Science and Technology	220 (21.8)	112 (18.6)	332 (20.6)	
Humanities and Social Sciences	604 (59.9)	491 (81.4)	1095 (68)	
GPA, n (%)				0.021^a^
1.50–1.99	11 (1.1)	7 (1.2)	18 (1.1)	
2.00–2.49	58 (5.8)	29 (4.8)	87 (5.4)	
2.50–2.99	169 (16.8)	127 (21.1)	296 (18.4)	
3.00–3.49	371 (36.8)	243 (40.3)	614 (38.1)	
3.50–3.99	389 (38.6)	196 (32.5)	585 (36.3)	
4.00	10 (1)	1 (0.2)	11 (0.7)	
Family history of mental illness, n (%)				0.008^a^
Yes	66 (6.5)	21 (3.5)	87 (5.4)	
No	942 (93.5)	582 (96.5)	1524 (94.6)	
Chronic illness, n (%)				0.065^a^
Yes	99 (9.8)	43 (7.1)	142 (8.8)	
No	909 (90.2)	560 (92.9)	1469 (91.2)	
Monthly income (Baht), n (%)				<0.001^a^
<5,000	652 (64.7)	466 (77.3)	1118 (69.4)	
5,001–10,000	320 (31.7)	131 (21.7)	451 (28)	
10,001–15,000	25 (2.5)	5 (0.8)	30 (1.9)	
>15,000	11 (1.1)	1 (0.2)	12 (0.7)	
High school type, n (%)				0.136^a^
Public school	709 (70.3)	445 (73.8)	1154 (71.6)	
Private school	299 (29.7)	158 (26.2)	457 (28.4)	
Residential area, n (%)				0.836^a^
Urban	373 (37)	185 (30.7)	558 (34.6)	
Rural	635 (63)	418 (69.3)	1053 (65.4)	
Stable partner, n (%)				0.01^a^
Yes	453 (44.9)	311 (51.6)	764 (47.4)	
No	555 (55.1)	292 (48.4)	847 (52.6)	
Close relationship, n (%)				< 0.001^a^
Alone	126 (12.5)	117 (19.4)	243 (15.1)	
Parents	263 (26.1)	255 (42.3)	518 (32.2)	
Friend	556 (55.2)	200 (33.2)	756 (46.9)	
Other family members	13 (1.3)	8 (1.3)	21 (1.3)	
Partner	50 (5)	23 (3.8)	73 (4.5)	
Faculty choice relevant to future career prospects, n (%)				0.436^a^
Yes	936 (92.9)	566 (93.9)	1502 (93.2)	
No	72 (7.1)	37 (6.1)	109 (6.8)	
Self-confidence, n (%)				0.004^a^
Low	166 (16.5)	64 (10.6)	230 (14.3)	
Medium	760 (75.4)	480 (79.6)	1240 (77)	
High	82 (8.1)	59 (9.8)	141 (8.8)	
Life satisfaction, n (%)				0.242^a^
Low	146 (14.5)	83 (13.8)	229 (14.2)	
Medium	422 (41.9)	231 (38.3)	653 (40.5)	
High	440 (43.7)	289 (47.9)	729 (45.3)	

a Chi-square test, p-value < 0.05; percentages are reported in parentheses.

Regarding health-related behaviors and lifestyle, most participants did not consume alcohol (69%) or smoke (93.7%), with 99.3% rejecting drug use. Regular exercise (three times a week) was reported by 50.2%, while 41.9% did not exercise. Approximately 62% reported getting sufficient sleep. More than half reported that their daily online activity ranged from 1 to 8 hours (66%). Most consumed non-healthy food (97.5%) and managed their time at a moderate level (52.2%), as presented in **Table 2**.

**Table 2:** Health behavior and lifestyle (n =1611).

Variable	Depression		Total	*P*-value
	No	Yes		
Alcohol intake, n (%)				0.567^a^
Yes	318 (31.5)	182 (30.2)	500 (31)	
No	690 (68.5)	421 (69.8)	1111 (69)	
Smoking, n (%)				<0.001^a^
Yes	62 (6.2)	39 (6.5)	101 (6.3)	
No	946 (93.8)	564 (93.5)	1510 (93.7)	
Substance use, n (%)				0.485^a^
Yes	8 (0.8)	3 (0.5)	11 (0.7)	
No	1000 (99.2)	600 (99.5)	1600 (99.3)	
Physical activity, n (%)				0.685^a^
No	421 (41.8)	254 (42.1)	675 (41.9)	
<3 times/week	503 (49.9)	306 (50.7)	809 (50.2)	
>3 times/week	84 (8.3)	43 (7.1)	127 (7.9)	
Sleeping adequacy, n (%)				<0.001^a^
Yes	583 (57.8)	414 (68.7)	997 (61.9)	
No	425 (42.2)	189 (31.3)	614 (38.1)	
Daily internet use (hours/day), n (%)				0.553^a^
<1	35 (3.5)	15 (2.5)	50 (3.1)	
1–8	657 (65.2)	406 (67.3)	1063 (66)	
9–16	268 (26.6)	150 (24.9)	418 (25.9)	
>17	48 (4.8)	32 (5.3)	80 (5)	
Dietary habit, n (%)				0.081^a^
Non-healthy	977 (96.9)	593 (98.3)	1570 (97.5)	
Healthy	31 (3.1)	10 (1.7)	41 (2.5)	
Time management, n (%)				0.136^a^
Low	167 (16.6)	78 (12.9)	245 (15.2)	
Medium	521 (51.7)	320 (53.1)	841 (52.2)	
High	320 (31.7)	205 (34)	525 (32.6)	

a Chi-square test, *p*-value <0.05; percentages are reported in parentheses.

The analysis and comparison of the proportions of variables associated with the presence or absence of depression, conducted using chi-squared tests in the sample group, revealed statistically significant differences between individuals with and without depression across the following domains: age (*p* = 0.008), university (*p* < 0.001), faculty (*p* < 0.001), GPA (*p* = 0.021), family history of psychiatric disorders (*p* = 0.008), monthly income (*p* < 0.001), having a partner (*p* = 0.01), close relationships (*p* < 0.001), self-confidence (*p* = 0.04), smoking (*p* < 0.001), and sleep adequacy (*p* < 0.001). Details of these findings are shown in **Tables 1**, **2**.

### 3.2 Prevalence of depression

**Figure 1:**
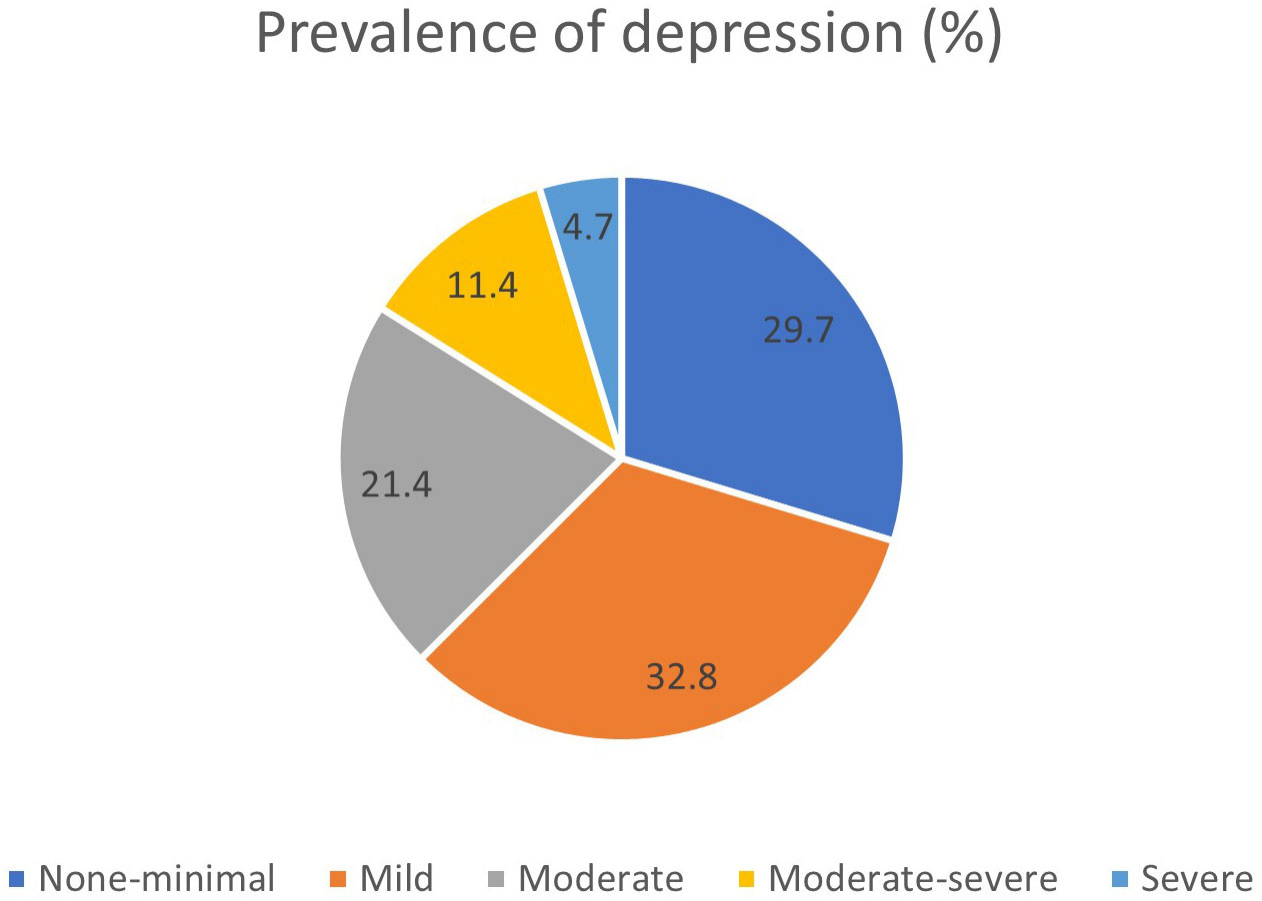
Prevalence of depression among students in Southern Thailand.

The overall prevalence of depression was 37.5%, with severity levels distributed as follows: moderate (21.4%), moderate to severe (11.4%), and severe (4.7%) (**Figure 1**).

### 3.3 Depressive symptoms

**Figure 2:**
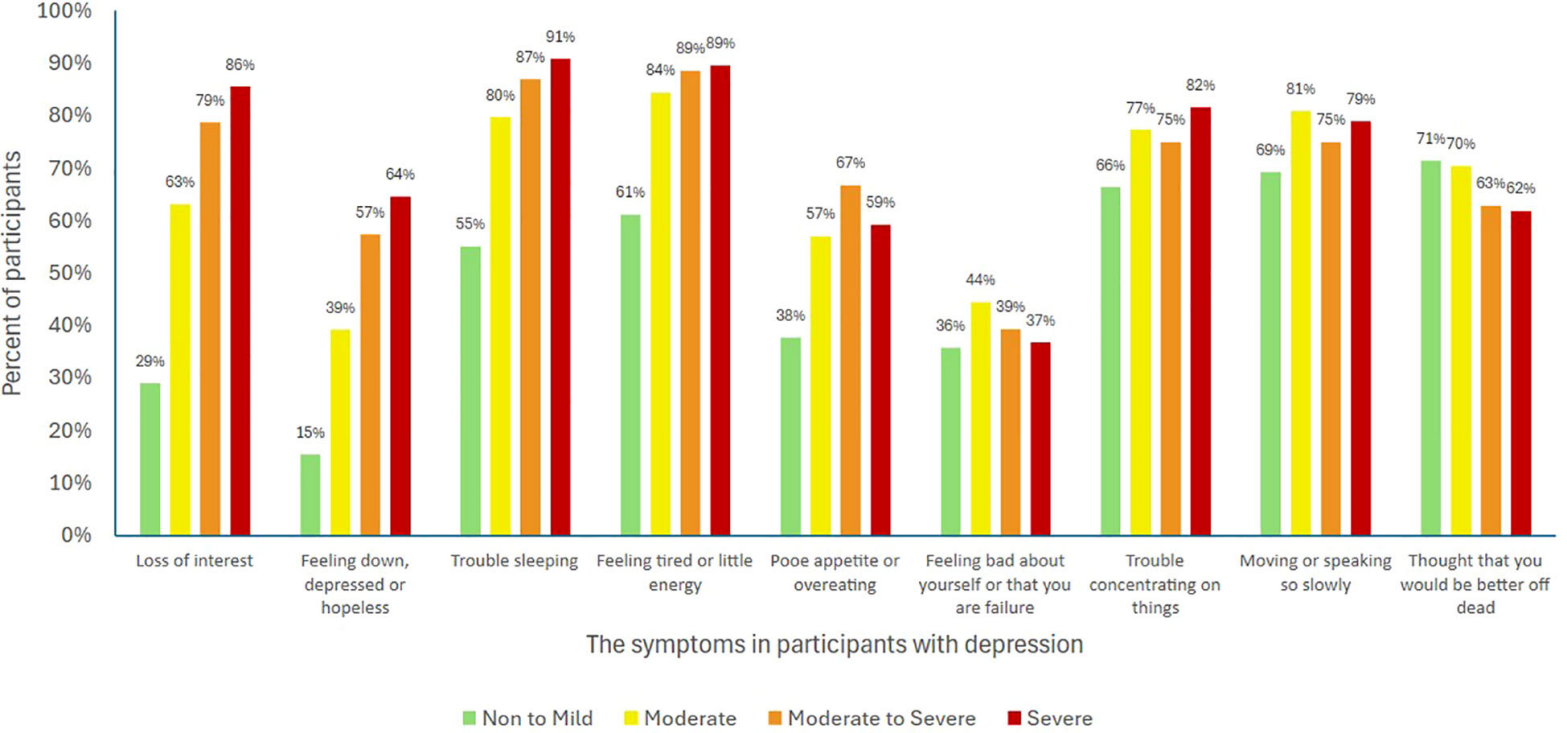
Depressive symptoms among students categorized by depression severity.

Data from the PHQ-9 questionnaire were analyzed based on the participants’ symptoms and the severity of depression. The results indicated that the most common symptom in the moderate severity group was feeling easily tired or lacking energy (84%), followed by speaking or doing things slowly enough for others to notice (81%). In the moderate to severe group, the most common symptom was tiring easily or lacking energy (89%), followed by difficulty sleeping or sleeping too much (87%). In the severe group, the most common symptom was difficulty sleeping or sleeping too much (91%), followed by tiring easily or lacking energy (89%) (**Figure 2**).

### 3.4 Factors associated with depression

Statistically significant factors related to depression included individuals with a GPA of 1.50–1.99, who had a 1.19 (95% CI 0.28–2.52) times higher likelihood of experiencing depression than those with a GPA of 2.00–2.49, and a 5.8 (95% CI 0.55–0.92) times higher likelihood of experiencing depression than those with a GPA of 4.00. Those who had attended a government secondary schools had a 1.4 (95% CI 0.55–0.92) times higher likelihood of experiencing depression than those who had attended private secondary schools. Those who were in a relationship had a 1.3 times higher likelihood (95% CI 0.6–0.99) of experiencing depression than those without a partner. Individuals with a close relationship with their family had a 2 times higher likelihood of experiencing depression (95% CI 0.37–0.65), and those with a close relationship with friends and partners had a 2.1 times higher likelihood (95% CI 0.22–0.87). Moreover, individuals who ate unhealthy food had a 2.3 times higher likelihood of experiencing depression than those who ate healthily (95% CI 0.2–0.96). Finally, those with moderate and high self-confidence had a 1.58 (95% CI 1.11–2.25) and 2.04 (95% CI 1.2–3.45) times higher likelihood of experiencing depression, respectively, than those with low self-confidence (**Table 3**).

**Table 3:** Logistic regression of factors associated with depression.

Variable	Crude OR (95% CI)	Adj. OR (95% CI)	*P* (Wald’s test)	*P* (LR-test)
GPA: (ref. = 1.50–1.99)				0.006
2.00–2.49	0.79 (0.28,2.24)	0.84 (0.28,2.52)	0.76	
2.50–2.99	1.18 (0.45,3.13)	1.59 (0.57,4.4)	0.373	
3.00–3.49	1.03 (0.39,2.69)	1.6 (0.59,4.37)	0.355	
3.50–3.99	0.79 (0.3,2.07)	1.17 (0.43,3.19)	0.764	
4.00	0.16 (0.02,1.51)	0.17 (0.02,1.79)	0.14	
High school type: Private vs. Public	0.84 (0.67,1.06)	0.71 (0.55,0.92)	0.009	0.009
Stable partner: No vs. Yes	0.77 (0.63,0.94)	0.77 (0.6,0.99)	0.044	0.044
Close relationship: (ref.= parents)				<0.001
Friend	0.37 (0.29,0.47)	0.49 (0.37,0.65)	<0.001	
Other family members	0.96 (0.71,1.3)	0.86 (0.61,1.21)	0.388	
Partner	0.63 (0.26,1.56)	0.78 (0.28,2.15)	0.632	
Alone	0.47 (0.28,0.8)	0.44 (0.22,0.87)	0.017	
Smoking: No vs. Yes	0.95 (0.63,1.43)	0.64 (0.37,1.08)	0.094	0.092
Substance use: No vs. Yes	1.6 (0.42,6.05)	3.5 (0.8,15.29)	0.096	0.083
Dietary habit: Healthy vs. Non-healthy	0.53 (0.26,1.09)	0.44 (0.2,0.96)	0.04	0.034
Self-esteem: (ref.=low)				0.014
Medium	1.64 (1.2,2.23)	1.58 (1.11,2.25)	0.012	
High	1.87 (1.2,2.9)	2.04 (1.2,3.45)	0.008	

OR, Odd ratio; 95% CI, 95% confidence interval; ref., reference; Adj., adjusted.

### 3.5 Accessing mental health services

According to the data on access to mental health services, most participants (83.6%) did not consider visiting a psychiatrist to be problematic. Furthermore, 92.6% of them did not consider seeing a psychiatrist as embarrassing either for themselves or for others, and 73.3% did not view the process of accessing mental health services in Thailand as complicated. However, 72.3% did not search for mental health information on the internet, from friends or teachers (90.5%) or from parents or guardians (90.4%). Regarding social support, 65% and 61.3% of participants did not believe that if they had mental health issues, their friends and teachers would encourage them to see a psychologist, doctor, or psychiatrist, respectively. Additionally, 57.1% were unaware that their university offered mental health counseling services (**Table 4**).

**Table 4:** Mental health service approach behavior categorized and stratified by depression.

Questions	Depression screening		Total	*P-*value
	Negative	Positive		
Total	1008	603	1611	
1. You feel that seeing a psychiatrist is complicated.				<0.001^a^
Never	943 (93.6)	403 (66.8)	1346 (83.6)	
Always	65 (6.4)	200 (33.2)	265 (16.4)	
2. You feel that seeing a psychiatrist is embarrassing (both for yourself and others).				<0.001^a^
Never	991 (98.3)	501 (83.1)	1492 (92.6)	
Always	17 (1.7)	102 (16.9)	119 (7.4)	
3. You feel that accessing mental health services in Thailand is a complex process.				<0.001^a^
Never	835 (82.8)	346 (57.4)	1181 (73.3)	
Always	173 (17.2)	257 (42.6)	430 (26.7)	
4. You often search for mental health information on the internet.				<0.001^a^
Never	853 (84.6)	312 (51.7)	1165 (72.3)	
Always	155 (15.4)	291 (48.3)	446 (27.7)	
5. You frequently ask friends or teachers for information about mental health.				<0.001^a^
Never	957 (94.9)	501 (83.1)	1458 (90.5)	
Always	51 (5.1)	102 (16.9)	153 (9.5)	
6. You often ask for mental health information from your parents or guardians.				0.015^a^
Never	934 (92.7)	523 (86.7)	1457 (90.4)	
Always	74 (7.3)	80 (13.3)	154 (9.6)	
7. If you were facing mental health issues, your friends would support you in seeking help from psychologists, physicians, and psychiatrists.				0.337^a^
Never	664 (65.9)	383 (63.5)	1047 (65)	
Always	344 (34.1)	220 (36.5)	564 (35)	
8. If you were experiencing mental health problems, your teachers might support you in seeking help from psychologists, physicians, and psychiatrists.				0.566^a^
Never	623 (61.8)	364 (60.4)	987 (61.3)	
Always	385 (38.2)	239 (39.6)	624 (38.7)	
9. You know that the university has mental health services.				0.427^a^
Never	568 (56.3)	352 (58.4)	920 (57.1)	
Always	440 (43.7)	251 (41.6)	691 (42.9)	

a Chi-square test, *p*-value < 0.05; percentages are reported in parentheses.

## 4 Discussion

The prevalence of depression among participants from the southern universities of the AUN-HPN in Thailand during the first three months of the academic year was 37.5%. This finding aligns with Luo et al. (22), who reported a 48.9% prevalence of depression among students in -19 countries, post-COVID. However, it was slightly lower than the 53.9% prevalence found by Starvaggi et al. (23) among students in Malaysia during the pandemic. By contrast, Kaewkanta and Rungreangkulkij (24) observed a lower prevalence of 31.9% among university students in Chiang Rai, Thailand, in 2015, before the COVID-19 pandemic. These trends indicate depression among students has not decreased and may even be increasing in the post-COVID-19 era.

This study did not identify any cases of depression among health science students, which contrasts with findings from the Psychiatric Association of Thailand reporting a 45.3% prevalence of depression among students in health science disciplines (25). It is possible that health science students possess greater knowledge and skills in managing stress and mental health issues, allowing them to prevent depression more effectively and to seek treatment or counseling before symptoms become pronounced. Alternatively, the small number of participants in this study may have reduced the likelihood of detecting depression, potentially underestimating its true prevalence. Furthermore, stigma surrounding mental health in health science fields may have led some students to conceal their depressive symptoms due to concerns about professional repercussions, resulting in a lower observed rate of depression than actually exists.

Students attending universities in the lower southern border region of Thailand reported a higher prevalence of depression than those in the upper southern region, which aligns with the findings of Lamaisaard et al. (9), who examined the prevalence of depression at Prince of Songkla University and found higher rates of depression among students at campuses in the three southern border provinces than among those at campuses in other southern provinces. These results suggest potentially greater mental health challenges faced by students in these regions.

Among participants with moderately severe depression, being easily fatigued or lacking energy, and speaking or doing things noticeably slowly may be “red flags” for developing depressive disorder. Regarding the non-mild severity group, these symptoms require monitoring as they may develop into moderate severity. Those with mild depressive symptoms should rest, relax, and exercise (26). For those with moderate to severe depression, symptoms of fatigue or lack of energy and difficulty sleeping, or excessive sleep were common. Individuals in this group should consult healthcare professionals such as general practitioners and counseling psychologists for initial support (27). Regarding the group with severe symptoms, the most common symptoms were difficulty sleeping or excessive sleep, and being easily fatigued or lacking energy, similar to those observed in the moderate severity group. For this group, consulting a doctor for an evaluation and mental health support to monitor and prevent the symptoms from worsening is crucial (28).

When analyzing depression-related factors, a low GPA score was a significant predictor. Individuals with lower GPAs demonstrated a higher risk of depression than those with higher GPAs. This finding aligns with that of Siddik et al. (29), who found that students with high GPAs exhibited greater confidence in their academic pursuits, potentially mitigating depressive symptoms. It is possible that individuals with higher GPAs may possess effective time management and planning skills, which help reduce stress and feelings of discouragement. They are also more likely to receive recognition and support from their families and instructors, which could lower their risk of academic failure. Furthermore, students who had attended private high schools have been found to exhibit a lower risk of depression than those who had attended public schools, as evidenced by Chekol et al. (30). Therefore, university personnel should closely observe mental health disparities, particularly among students with lower GPAs and those coming from public high schools. Private schools often offer better facilities, such as a more conducive learning environment, and higher-quality nutrition and health services. Some schools also provide accessible psychologists or guidance counselors, which enhance students’ access to adequate care and support.

Another factor associated with depression risk was relationship status. Individuals without a romantic partner exhibited a lower risk of depression than those who were in relationships. This finding contrasts with the findings of Ramón-Arbués et al. (31), who suggested that the absence of a stable partner increases the risk of depression. Being in a romantic relationship often comes with certain expectations, such as the desire for care, understanding, and stability. When these expectations are not met, even within ostensibly positive relationships, individuals may experience disappointment, anxiety, and an increased risk of developing depressive symptoms. Furthermore, the quality of interpersonal relationships has been shown to influence depression risk, with strong social bonds with friends and partners associated with lower risk of depression. This aligns with Pandi (32), who found that students with close friendships demonstrated lower average depression scores than those who avoided social interactions with peers. Positive relationships foster a sense of closeness with friends and romantic partners. Being accepted for who one is and experiencing understanding from others can reduce feelings of loneliness. Moreover, such relationships provide opportunities for open communication and emotional expression, which can facilitate better stress management and problem-solving.

Dietary habits also exert a significant influence on depression. Individuals who consumed healthy foods exhibited a lower likelihood of experiencing depression than those who consumed unhealthy foods. This observation aligns with the findings of Anosike et al. (33), who demonstrated a relationship between diet type and depression. Furthermore, Molendijk et al. (34) found that high-quality diets were associated with a reduced risk of depression. These findings highlight the importance of incorporating food literacy into psycho-education programs to prevent worsening of depression levels among university students. Healthy foods such as vegetables, fruits, and healthy fats have a positive effect on neurotransmitters associated with mood regulation. They also help reduce brain inflammation, which is one of the underlying mechanisms linked to depression (35).

Self-confidence is another factor influencing depression. This study found that a higher level of self-confidence was associated with a greater risk of depression, which contrasts with Lun et al.’s (17) study, which identified low self-confidence as a contributing factor to depression. Individuals with high self-confidence may have unrealistically high expectations for themselves, leading to disappointment if they fail to meet their goals. Additionally, such individuals may fail to acknowledge or express feelings of disappointment, stress, or emotional vulnerability, preventing them from receiving the appropriate support or help and increasing the risk of long-term depression.

Regarding the data on accessing mental health services, most participants had a positive attitude and believed that they could seek help if needed. Nearly half of the participants with depression sought mental health information online. While this can encourage treatment-seeking, it also has drawbacks, such as the possibility of receiving incorrect information. Starvaggi et al. (23) found erroneous information to be prevalent on social media, which may prevent individuals with depression from receiving the correct treatment and could be harmful. More than half of the participants in the current study chose not to inquire about mental health information from close contacts, such as parents, guardians, teachers, and friends, which may be owing to some groups perceiving this as stigma. Tangjitboonsanga and Charnsil (36) found higher levels of perceived stigma among the Thai population, often rooted in cultural beliefs. This negative attitude can lead to various unhealthy behaviors. Some individuals may worry that those who listen will be concerned, fear gossip, or think that the listener may not understand their true condition, believing it to be normal rather than a disorder. Consequently, they may prefer talking to strangers or seeking information themselves, which may lead to encountering incorrect information.

The participants also exhibited varying levels of awareness regarding whether their university offered mental health consultation services. Limited awareness may prevent them from utilizing these services, leaving their conditions untreated. Therefore, increasing public awareness of depression, initial mental health screening, and strategies for assessing and preventing mental health disorders should be prioritized.

### 4.1 Study limitations and suggestions

This study employed a cross-sectional design, which enabled the identification of factors associated with depression but did not allow for the establishment of causal relationships. Future studies should consider employing a prospective cohort design to explore causal relationships more effectively. The sample was limited to first-year students, excluding upper-year students who may face different challenges. Furthermore, using self-report surveys can lead to response bias, especially due to stigma, and the study did not fully address cultural attitudes toward mental health. Although the PHQ-9 is a widely validated screening tool, it does not provide accurate clinical diagnoses. Future research should include clinical interviews for better results. Finally, qualitative research is recommended to examine why individuals with depression perceive mental health services as complicated to access. Such studies could provide deeper insights into their experiences.

## 5 Conclusion

The prevalence of depression among first-year students at universities in the southern region of Thailand after the COVID-19 pandemic was 37.5%. Protective factors associated with lower levels of depression included a higher GPA score, having attended a private secondary school, being single, having close relationships, and proper nutrition. Conversely, the risk factor for depression was higher self-confidence. Common symptoms across moderate, moderate to severe, and severe depression groups included difficulty falling asleep or excessive sleeping and fatigue, or lack of energy. These symptoms should be closely monitored due to their strong association with depression. Among the students with and without depression, most participants considered accessing services in Thailand as not being complicated. The majority preferred to seek mental health information online rather than asking individuals close to them, such as parents, guardians, teachers, or friends. Additionally, more than half were unaware that their university provided mental health services. Therefore, to prevent depression among students, universities should focus on early screening, increase awareness of available mental health services, and promote their accessibility.

## Data Availability

The original contributions presented in the study are included in the article/supplementary material. Further inquiries can be directed to the corresponding author.
